# DG2GAN: improving defect recognition performance with generated defect image sample

**DOI:** 10.1038/s41598-024-64716-y

**Published:** 2024-06-26

**Authors:** Fuqin Deng, Jialong Luo, Lanhui Fu, Yonglong Huang, Jianle Chen, Nannan Li, Jiaming Zhong, Tin Lun Lam

**Affiliations:** 1 School of Mechanical and Automation Engineering, The Wuyi University, Jiangmen, 529000 China; 2 School of Computer Science and Engineering, Faculty of Innovation Engineering, Faculty of Innovation Engineering, Macau University of Science and Technology, Macau, 999078 China; 3 Shenzhen Huatuo Semiconductor Technology Co, LTD, Shenzhen, China; 4 Shenzhen Institute of Artificial Intelligence and Robotics for Society, School of Science and Engineering, The Chinese University of Hong Kong, Shenzhen, China

**Keywords:** Data augmentation, Defect image generation, Generative adversarial network (GAN), Deep learning, Surface defect classification, Energy science and technology, Engineering, Materials science, Mathematics and computing, Physics

## Abstract

This article aims to improve the deep-learning-based surface defect recognition. In actual manufacturing processes, there are issues such as data imbalance, insufficient diversity, and poor quality of augmented data in the collected image data for product defect recognition. A novel defect generation method with multiple loss functions, DG2GAN is presented in this paper. This method employs cycle consistency loss to generate defect images from a large number of defect-free images, overcoming the issue of imbalanced original training data. DJS optimized discriminator loss is introduced in the added discriminator to encourage the generation of diverse defect images. Furthermore, to maintain diversity in generated images while improving image quality, a new DG2 adversarial loss is proposed with the aim of generating high-quality and diverse images. The experiments demonstrated that DG2GAN produces defect images of higher quality and greater diversity compared with other advanced generation methods. Using the DG2GAN method to augment defect data in the CrackForest and MVTec datasets, the defect recognition accuracy increased from 86.9 to 94.6%, and the precision improved from 59.8 to 80.2%. The experimental results show that using the proposed defect generation method can obtain sample images with high quality and diversity and employ this method for data augmentation significantly enhances surface defect recognition technology.

## Introduction

In the manufacturing industry, defect recognition plays a crucial role in ensuring product quality and optimizing production processes. Traditional manual defect detection methods are time-consuming, labor-intensive, and susceptible to human interference^1^. Automated visual defect detection emerges as an effective solution to address these issues. However, when employing automated defect detection, two primary challenges arise: First, there exists a significant imbalance between non-defective and defective data. This imbalance significantly skews the distribution of detection data, thereby limiting the performance of defect recognition models^2^. Second, the lack of data diversity, prevents the defect dataset from comprehensively representing the distribution of defect characteristics, thereby affecting the robustness of recognition models. Therefore, how to augment a large amount of balanced and diverse data is a prerequisite for automated visual defect detection^3^.

Traditional methods for generating defect images include manually damaging defect-free images and computer modeling. For instance, Parlak et al.^4^ used traditional methods to damage the surface of aluminum castings and other methods to study aluminum casting surface defects, while Jolly et al.^5^ employed computer modeling techniques to create defects in aluminum castings to improve the quality of aluminum castings. Additionally, Shorten et al.^6^ mentioned the improvement of epoxy resin images lacking defect occurrences through methods like cropping, rotation, flipping, and translation. However, these traditional methods are only suitable for simple defects, as the generated data fails to meet expectations in terms of diversity and quality for data augmentation. Moreover, methods such as rotation, scaling, translation, cropping, etc., for data augmentation are often only applicable to specific detection and recognition scenarios.

Currently, in the field of data augmentation, the primary methods for image generation using deep learning include Generative Adversarial Networks (GANs)^7^ and Variational Autoencoders (VAEs)^8^. These methods have made significant strides in recent years in the field of image synthesis technology, but they also encounter several challenges. Firstly, they all require a large amount of training data. However, obtaining a large-scale sample of defects in the manufacturing industry is extremely challenging, especially considering the scarcity of defective samples in industrial manufacturing, leading to an imbalance between defect-free and defective data. Secondly, original GANs often generate relatively simple structures and textures, limiting their ability to synthesize complex, irregular defects with significant random variations. Although high-quality images can be generated through a large amount of data, diversity is lacking. Thirdly, the image quality synthesized by VAEs may be unstable and unrealistic^9^. Although introducing additional noise can generate diverse defect images, this noise may render the generated images unrealistic, causing irrelevant transformations that compromise the authenticity of the data and result in poor image quality.

In recent years, many methods have been proposed to address the aforementioned issues, among which cycle-consistent networks have shown some progress^10^. Different from introducing noise to generate images, this method utilizes a large volume of non-defective images to produce defective images. Based on the transformation of characteristics from both sides, it tackles the problem of requiring an extensive dataset for training the generation model. Through the cycle consistency loss, the generated defect images are produced by feature transformation between non-defective and defective domains, addressing the issues of insufficient defect data and imbalanced raw data, avoiding the introduction of irrelevant transformations that compromise data authenticity.

To address the issues of imbalance, lack of diversity, and poor quality in the dataset, this paper proposes a novel generation method called DG2GAN. Based on image feature transformation utilizing cycle consistency loss, it generates defect images from a large number of defect-free images, thereby resolving the problem of requiring a significant amount of defect data for training the generation model. To increase the diversity of generated defect images, DJS (Jensen-Shannon divergence)^11^ is introduced in the added discriminator loss. It measures the difference between two probability distributions and optimizes the methods of these differences. By controlling the overlap of feature distributions between the generated sample distribution Pg and the real sample distribution Pr, it enhances the diversity of generated images. To maintain the diversity of generated images while ensuring that the quality meets the requirements of data augmentation, a new DG2 adversarial loss was proposed in DG2GAN. This ensures that the generated defect images retain high quality and diversity. Additionally, this paper designs an evaluation method to assess the comprehensive performance of generated images, including both quality and diversity. Experimental results demonstrate that augmenting the training set of classification recognition models with generated data effectively improves classification performance. This paper contains the following three contributions.

- To address the issue of insufficient quantity and imbalanced defect image samples in surface defect detection tasks, a defect image generation method called DG2GAN with DG2 adversarial loss is proposed.
- The incorporation of DJS into the augmented discriminator, which optimizes image feature distributions, assists the generation model in preserving sample quality while enhancing sample diversity.
- The DG2GAN generation method is employed to augment high quality and highly diverse data, enhancing the detection performance of the surface defect detection model and facilitating the success of surface defect detection tasks.

The rest of this essay is structured as follows. “Related work” provided a concise summary of the relevant work in surface defect recognition and defect image generation. “Methods” introduces a novel GAN framework based on image transformation, named DG2GAN, outlining its content and related work. “Experiment” validates the effectiveness of the generated defect images using the evaluation protocol of defect recognition networks and associated experiments. It discusses how this method aids in improving the detection accuracy of surface defect detection models. “Conclusion” summarizes all the work of this paper.

## Related works

Traditional methods for defect identification include manual visual inspection and image processing-based approaches. Manual inspection is susceptible to subjective judgments by inspectors, which may lead to inconsistent and inaccurate results due to factors such as fatigue, experience, emotions, among others^12^. Additionally, traditional image processing methods, such as histogram statistics, Grey-Level Co-occurrence Matrix (GLCM), and Fourier features, necessitate manual design and selection of features, which is laborious, lacks reusability, and is challenging to adapt to diverse and complex defect scenarios. These methods have limited capability in identifying defect regions that are small, blurry, have low contrast, or are subjected to significant interference, consequently restricting the performance of the identification model. Sironi et al.^13^ achieved efficient and robust feature extraction and classification using histograms, but the acquisition and processing of data require specialized equipment and technical support. Lappas et al.^14^ achieved anomaly detection by extracting frequency domain features using Fourier transform and encoding and decoding with autoencoders. However, strong data dependency remains a major challenge. Moreover, Benco et al.^15^ proposed a method for extracting color and texture information using GLCM to achieve classification recognition. However, it performs poorly when handling complex data and requires a large amount of data support. However, these methods are limited in their ability to identify complex defects due to the limitations of the data.

Deep learning methods have been widely adopted in defect detection, resolving some challenges faced by traditional defect detection methods. Currently, commonly used deep learning network architectures include AlexNet^16^, VGG^17^, ResNet^18^, DenseNet^19^, SENetV2^20^, ShuffleNet^21^ and others. These network models are frequently utilized for defect detection tasks, and there are also specialized detection networks designed for specific task requirements. Hou et al.^22^ designed a new CNN detection network that automated the detection of fabric surface defects, reducing the workload of manual inspection. Niu et al.^23^ used the VGG network to identify and classify defects synthesized using generative adversarial network (GAN) methods. Liang et al.^24^ proposed a ShuffleNetV2-based approach to classify defects in complex backgrounds such as ink codes on bottles. Zhang et al.^25^ utilized commonly used image recognition models like ResNet and DenseNet to detect defects in augmented data. Alam et al.^26^ used the ResNet18 network to identify defects in epoxy drop images synthesized using a variant of CycleGAN. Despite the significant advantages of deep learning in surface defect detection, its training requires a large number of defect image samples^27^. The limited quantity of defect samples in practical production has always been a challenge in using deep learning models for defect detection^28^. Therefore, it is necessary to design a defect generation method that meets the task requirements to address issues such as imbalanced and insufficient defect data, lack of diversity, and poor quality. This approach aims to expand the dataset and assist in enhancing the defect detection performance of deep learning models.

In current research, some methods aim to generate high-quality defect images. Hu et al.^29^ introduced the Relative Mean Generative Adversarial Network (TARGAN) to enhance the quality of defect images. Due to data scarcity and severe imbalance among different defect patterns, Branikas et al.^30^ employed a novel data augmentation technique based on CycleGAN to expand the dataset. Wang et al.^31^ proposed a CycleGAN-based data augmentation method, facilitating data expansion through mapping between glyph image data domain and real sample data domain. Furthermore, Alam et al.^26^ presented a data augmentation method for synthesizing defect epoxy droplet images, optimizing the cycle consistency loss function using a CycleGAN variant to generate high-quality defect epoxy droplet images.

Nguyen et al.^32^ proposed D2GAN to address the issue of diversity in generating defect images. They achieved this by incorporating a diverse reward mechanism in the discriminator to encourage diverse image generation by distinguishing between generated and real features. Liu et al.^33^ proposed the Dual Discriminator Wasserstein Generative Adversarial Network (D2WGAN), optimizing the generation model using KL divergence and reverse KL divergence to enhance diversity, generating a rich variety of samples. Zhang et al.^25^ introduced DefectGAN, which generates diverse defect images by introducing noise during the encoding and decoding processes. However, the generated defect images might include completely imperceptible or potentially undesirable samples. Furthermore, Niu et al.^23^ proposed the Surface Defect Generative Adversarial Network (SDGAN), which encourages the generation of diverse features by adding a discriminator to the network architecture.

In the above methods, various image generation techniques have their advantages and limitations depending on the requirements of different tasks and the characteristics of the data. Some methods focus on improving the quality of generated images by employing image transformation models, but they often lack diversity. On the other hand, some methods emphasize the diversity of generated images, which may compromise the authenticity of the generated images, especially in cases of limited data and imbalance, resulting in issues such as poor image quality and insufficient diversity in the generated images. Therefore, this paper proposes a new image transformation method, namely DG2GAN. By introducing the proposed DG2 adversarial loss and incorporating DJS-optimized loss in the added discriminator, we ultimately combine it with cycle consistency loss to generate defect images from a large number of defect-free images. This enables the DG2GAN model to generate high-quality and diverse defective images while avoiding the introduction of irrelevant transformations that may compromise the authenticity of the data, thus enhancing defect detection performance through data augmentation.

## Methods

**Figure 1:**
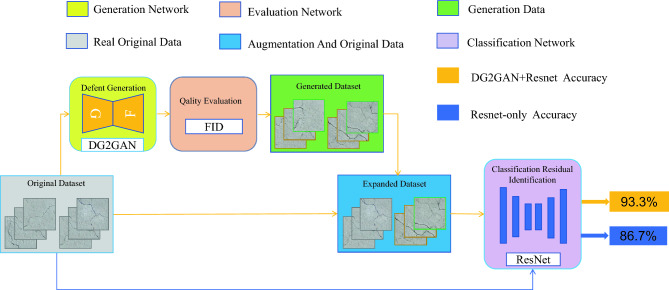
Program of the proposed approach.There are three components: defect generation, quality evaluation, and defect recognition.

We propose a new framework named DG2GAN. As shown in Fig. 1, the work in this paper consists of three main parts: defect generation, quality assessment and defect classification identification.

- *Defect generation*: We propose a defect generation method called DG2GAN. It utilizes cycle consistency loss to generate defect images from a large number of defect-free images. We introduce DJS-optimized added discriminator loss to encourage the generation of diverse defect images. We design DG2 adversarial loss to ensure the generation of high-quality and diverse defect images.
- *Quality evaluation*: We utilize the FID (Fréchet Inception Distance) evaluation method to assess and compare the comprehensive performance of defect images generated by DG2GAN with those generated by other advanced and related generation methods.
- Defect Classification Recognition: We divide the original data and augmented data into two sets and input them into the ResNet network for training, and compared the ResNet network training results using only the original data and the extended data to evaluate the impact of the extended data on the defect classification and recognition.

### Defect image generation

**Figure 2:**
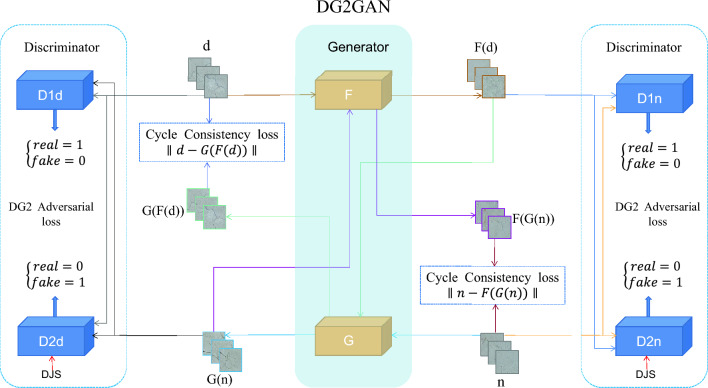
Architecture of the DG2GAN, which contains six networks: two generators and four discriminators. The objective of DG2GAN includes DG2 adversarial loss, cycle consistency loss, and DJS.

In addressing the problem of data imbalance and the scarcity of data in practical production, leading to a decrease in defect detection accuracy and precision, this paper aims to utilize the DG2GAN generation method to augment existing limited data. The goal is to generate high-quality and diverse effective data, thereby enhancing the performance of defect detection. Our approach also demonstrates that introducing DJS-optimized discriminators can enhance the quality and diversity of generated images. In light of this, we propose the DG2GAN model, the structural diagram of which is illustrated in Fig. 2.

DG2GAN consists of six networks: two generators, namely *F* and *G*, and four discriminators, namely *D*1*n*, *D*1*d*, *D*2*n*, and *D*2*d*. In this, generator *F* transforms real defective images *d* into artificially generated defect-free images *F*(*d*), while generator *G* creates fake defective images *G*(*n*) from real defect-free images *n*. This segment constitutes the transformation for the cycle consistency loss network, making full use of both defect-free and defective data. Discriminators *D*1*n* and *D*2*n* primarily differentiate between defect-free images *n* and the generated defect-free images *F*(*d*). On the other hand, discriminators *D*1*d* and *D*2*d* specifically distinguish between defective images *d* and the generated defective images *G*(*n*). Due to the reward and penalty mechanisms of *D*2*d* and *D*2*n* in the network structure, DJS optimization is introduced to facilitate the generation of more diverse defective images. In discriminators *D*1*n* and *D*1*d*, their task is to recognize real distribution features and generated distribution features, making the generated image features closer to real image features. In discriminators *D*2*n* and *D*2*d*, rewarding the distribution features of generated images greater than those of real images encourages the generation of diverse features. The role of the four discriminators gives rise to the DG2 adversarial loss, ensuring the generation of high-quality and diverse images. Our optimization objectives encompass three aspects: cycle consistency loss, DJS optimized discriminator loss, and DG2 adversarial loss.

**Cycle consistency loss** The cycle consistency loss is a crucial component of DG2GAN, addressing the imbalance between defect and defect-free samples and the limited quantity of defect samples in production. Typically, defect and defect-free data exhibit similar characteristics, with the majority of regions in defect images resembling defect-free images except for the defective areas. Therefore, the optimal approach is to generate defective images based on non-defective images, preserving data consistency and rationality while avoiding the introduction of unrelated transformations that could compromise the authenticity of the data. In our DG2GAN model, Cycle Consistency Loss offers the following three advantages.

1 $$\begin{aligned} L_{cycle}(G,F)&= E_{(n\in P_{r}(n) )}\left\| F(G(n))-n \right\| _{1} +E_{(d\in P_{r}(d) )}\left\| G(F(d))-d \right\| _{1} \end{aligned}$$

Firstly, by fully leveraging production data from industrial sites and generating defect images from defect-free images, we address the issues of imbalanced original data and insufficient quantity of defect samples. Second, it maintains the consistency and rationality of the data by generating new defective images through the transformation of features from both the non-defective and defective domains. This approach avoids introducing unrelated transformations that could compromise the authenticity of the data. Third, it aids model convergence, as the cycle consistency loss helps the model converge when dealing with imbalanced or limited data.

**DJS optimized discriminator loss** The introduction of DJS auxiliary classification in the added *D*2*n* and *D*2*d* discriminator loss functions aims to improve the diversity of generated features. By controlling the relative importance of $DJS(P_{g}||P_{r})$ in the discriminator loss, the generation model is encouraged to produce more diverse feature distributions. This helps prevent the generator from falling into a single mode or repeatedly generating similar samples, thereby increasing the diversity of generated samples.

The introduction of DJS as an additional optimization objective in the generation method aims to increase the diversity of samples while maintaining sample quality. However, in the actual optimization process, it is necessary to balance DJS with other objective functions to achieve better generation results. The specific setting of weight parameters and tuning methods usually require experimentation and adjustment based on the specific data situation. In SDGAN, $DJS(P_{g}||P_{r})$ is used as an optimization loss for the generator to increase the diversity of generated images. Although optimizing the generator loss through DJS improves the diversity of generated defect images, it may lead to the generation of some meaningless samples. Chengtao et al.^34^ proposed a method that focuses on optimizing the discriminator’s discriminative loss by introducing DJS as a supplementary optimization objective. This approach aims to ensure an overlap between the generated feature distribution and the real feature distribution on the support set. This control facilitates the optimization of both the quality and diversity of generated defect images, thereby enhancing the diversity of generated images.

We utilize DJS as a supplementary optimization objective on the discriminators added in DG2GAN. Unlike SDGAN, which pairs it with the generator for optimization, and unlike Chengtao et al.^34^, who applied DJS optimization to all discriminators, in DG2GAN, we use DJS as a supplementary optimization objective specifically for the added *D*2*n* and *D*2*d* discriminators. First, in the initialization method of the discriminator network model, new trainable parameters are defined. The network can automatically adjust these parameters during training to optimize the loss function. Secondly, during the forward propagation process, the discriminator not only returns the traditional discrimination results but also computes and returns the DJS loss, providing an additional optimization objective for the training process. This adjustment makes the loss function more adaptive to the data distribution, improving the overall performance of the Generative Adversarial Network (GAN). Lastly, by using DJS to optimize the discrimination loss for *D*2*n* and *D*2*d*, where the reward for the real feature distribution is less than the reward for the generated feature distribution, the generator needs to produce different types of samples to deceive the discriminator when facing different discriminators. This can achieve training diversity. This adversarial approach enhances the generator’s capability, making the generated samples more diverse and realistic. Experimental results demonstrate that the introduction of DJS optimization in the DG2GAN model helps enhance the overall performance of generating defect images. This improvement is characterized by both quality and diversity enhancements, along with a reduction in the generation of meaningless defect samples.

The DJS optimized discriminative loss for *D*2*d* is formulated as follows:

2 $$\begin{aligned} L_{D2d}&=DJS\times \mu + E_{(d\in P_{r}(d) )}[\log {D2d(d)}] +E_{(n\in P_{r}(n) )}[\log {(1-D2d(G(n)))} ] \end{aligned}$$

Equation (2) combines the discriminative loss with DJS optimization. The weight $\mu$ of DJS in this equation represents the relative importance of DJS in influencing the discriminative loss and can be used to adjust the overlap between the generated feature distribution and the real feature distribution on the support set. Unlike the reward-penalty mechanism of the D1d discriminator, DJS gives higher rewards to generated images and smaller rewards to real images. By minimizing DJS, the entire discriminative loss is minimized. As the discriminative mechanism of *D*2*d* tends to favor diverse features, the collaborative action of the generator and discriminator encourages the generation of more diverse defect images.

**DG2 adversarial loss** In the two D1 discriminators, the reward for the real feature distribution is greater than the reward for the generated feature distribution, encouraging the generated images to deceive the discriminator and generate images closer to the original features. In the two D2 discriminators, a higher reward is given to the generated image feature distribution, contrary to the reward mechanism of D1. DG2 adversarial loss is proposed by setting up multiple discriminator losses.

For the generator *G* and its discriminator *D*1*d*, the function of the adversarial loss is expressed as follows:

3 $$\begin{aligned} L_{gan1}(G;D1d;n;d)&= E_{(d\in P_{r}(d) )}[\log {D1d(d)}] +E_{(n\in P_{r}(n) )}[\log {(1-D1d(G(n)))} ] \end{aligned}$$

Equation (3) describes the adversarial loss between the generator G and the discriminator *D*1*d*. The objective of the generator *G* is to produce defect images *G*(*n*) similar to the defect domain *d*. The adversarial loss is computed between the predicted results of the generated fake samples on *D*1*d* and the target labels. This encourages the generator *G* to generate images that can deceive the discriminator *D*1*d*. The task of discriminator *D*1*d* is to distinguish between the generated defect images *G*(*n*) and the real defect images *d*, even if *D*1*d*(*d*) tends to 1, and *D*1*d*(*G*(*n*)) tends to 0. The goal of generator *G* and discriminator *D*1*d* is to generate images that are close to the original data and capable of deceiving the discriminator through adversarial training.

For the newly added discriminator *D*2*d*, its role in the generator *G* and the loss function are as follows:

4 $$\begin{aligned} L_{gan2}(G;D2d;n;d)&= E_{(n\in P_{r}(n) )}[\log {D2d(G(n))}] +E_{(d\in P_{r}(d) )}[\log {(1-D2d(d))} ] \end{aligned}$$

The introduction of multiple discriminators, particularly the addition of the *D*2*d* discriminator compared to *D*1*d*, aims to provide more detailed guidance and supervision for the generator. This configuration enables the generator to receive more precise guidance in different tasks. According to the D2 adversarial loss concept introduced by the added discriminator, it helps enhance the diversity of generated defect images. The loss for real and generated samples on the second discriminator is described by Eq. (4). In comparison to *D*1*d* and *D*1*n*, *D*2*n* and *D*2*d* provide a higher reward for the generated images and a lower reward for real images. In other words, when *D*2*d*(*G*(*n*)) approaches 1 and *D*2*d*(*d*) approaches 0, it encourages the generated diverse samples to deceive *D*2*d*, thereby increasing the diversity of generated defect images.

The DG2 adversarial loss proposed by the interaction of *D*1*d* and *D*2*d* with the generator *G* is expressed as follows:

5 $$\begin{aligned} L_{DG2gan}(G;D2d;n;d)&= \lambda L_{gan1}(G;D1d;n;d) +\beta L_{gan2}(G;D2d;n;d) \end{aligned}$$

Equation (5) represents the DG2 adversarial loss, considering the performance of the generator *G* and *F* on *D*1*d*, *D*1*n*, *D*2*n*, and *D*2*d*. It balances the adversarial losses in two different directions, guiding the generator to retain the original feature distribution information in the defect-free area while producing diverse defect features through learning from the original features in the defective area. This aims to find the optimal balance point for better generation performance. $\lambda$ controls the relative importance of generating high-quality images, with $L_{gan1}$ ensuring the generation of a defect dataset with high image quality. $\beta$ controls the relative importance of generating diversity in defect images, with $L_{gan2}$ ensuring the generation of a diverse defect dataset. Therefore, the DG2 adversarial loss, through the weights of $\lambda$ and $\beta$, controls the generation of image quality and diversity. Considering different loss components comprehensively helps the generator and discriminator be more robust in various situations.

**Full objective** The complete objectives of DG2GAN include both DG2 adversarial loss and cycle consistency loss, as represented by Equation (6).

6 $$\begin{aligned} \begin{aligned} L_{DG2GAN}(G;F;D1_{d} ;D1_{n};D2_{d} ;D2_{n}) =&L_{DG2gan}(G;D2d;n;d)+L_{DG2gan}(F;D2d;n;d) \\&+ L_{cycle}(G,F) \end{aligned} \end{aligned}$$

Firstly, cycle consistency loss facilitates one-to-one feature transformation from defect-free images to defect images, effectively utilizing imbalanced data to generate defect images while avoiding the introduction of irrelevant transformations that compromise data authenticity. Secondly, the introduction of DJS optimization in the additional *D*2*n* and *D*2*d* discriminators enhances the diversity of generated images by optimizing the discriminator loss. Finally, the overall loss in Eq. (6) comprehensively considers different loss terms, forming the ultimate DG2GAN loss. This comprehensive loss function allows the generator and discriminator to more realistically present non-defective regions during adversarial training, generating defect features that are diverse and rich, ultimately producing high-quality and diverse images.

### Evaluation protocol for generative models

The selection of evaluation methods for generative models has always been a challenging task^35^, especially when seeking methods that are applicable for both human visual assessment and quantitative measurement. FID (Fréchet Inception Distance)^36^ was one commonly employed method, serving as a crucial metric for assessing the comprehensive performance of GAN-generated images. It quantifies the Gaussian distribution distance in the feature space between generated images and real images. FID focuses on the feature distribution between generated images and real images, aiding in effectively capturing the overall quality and authenticity of generated images. Additionally, to some extent, it reflects the diversity of generated images.

7 $$\begin{aligned} FID(P_{r} ;P_{g})&= \left\| \mu _{r}- \mu _{g} \right\| +Tr(C_{r}+C_{g}-2(C_{r}C_{g}))^{1/2} \end{aligned}$$

FID measures the distance between the real distribution and the generated distribution, indicating the difference in feature distribution between generated and real images. A lower FID value suggests that the feature distribution of generated images is closer to that of real images. Therefore, lower FID scores indicate better overall performance of generated images. In the formulas, $\mu _{r}$ and $\mu _{g}$ represent the mean values of features in real and generated images, while $C_{r}$ and $C_{g}$ represent the covariance values of features in real and generated images. In this paper, FID is utilized as an evaluation metric for assessing the generated images.

### Defect recognition network

Defect classification recognition can be divided into original data classification recognition and augmented data classification recognition, where augmented data includes both original and enhanced data. The classification recognition is performed using the ResNet network model. Without modifying the main network structure of ResNet, the input image size is kept at 256$\times$256$\times$3. The modification involves reducing the number of neurons in each layer of the fully connected layer by one sequence. The last layer outputs two categories, representing the defect-free and defect classes in the dataset. Experimental results demonstrate that the defect dataset generated by our proposed method is an effective augmentation technique, leading to improved accuracy in defect detection classification recognition. In the ResNet model, the ADAM solver^37^ is employed as the optimization method, and dropout^38^ is used to enhance the robustness of the defect classification model. The learning rate is set to 0.0002, and the model is trained for 1000 epochs.

## Experiments and analysis

### Dataset

**Figure 3:**
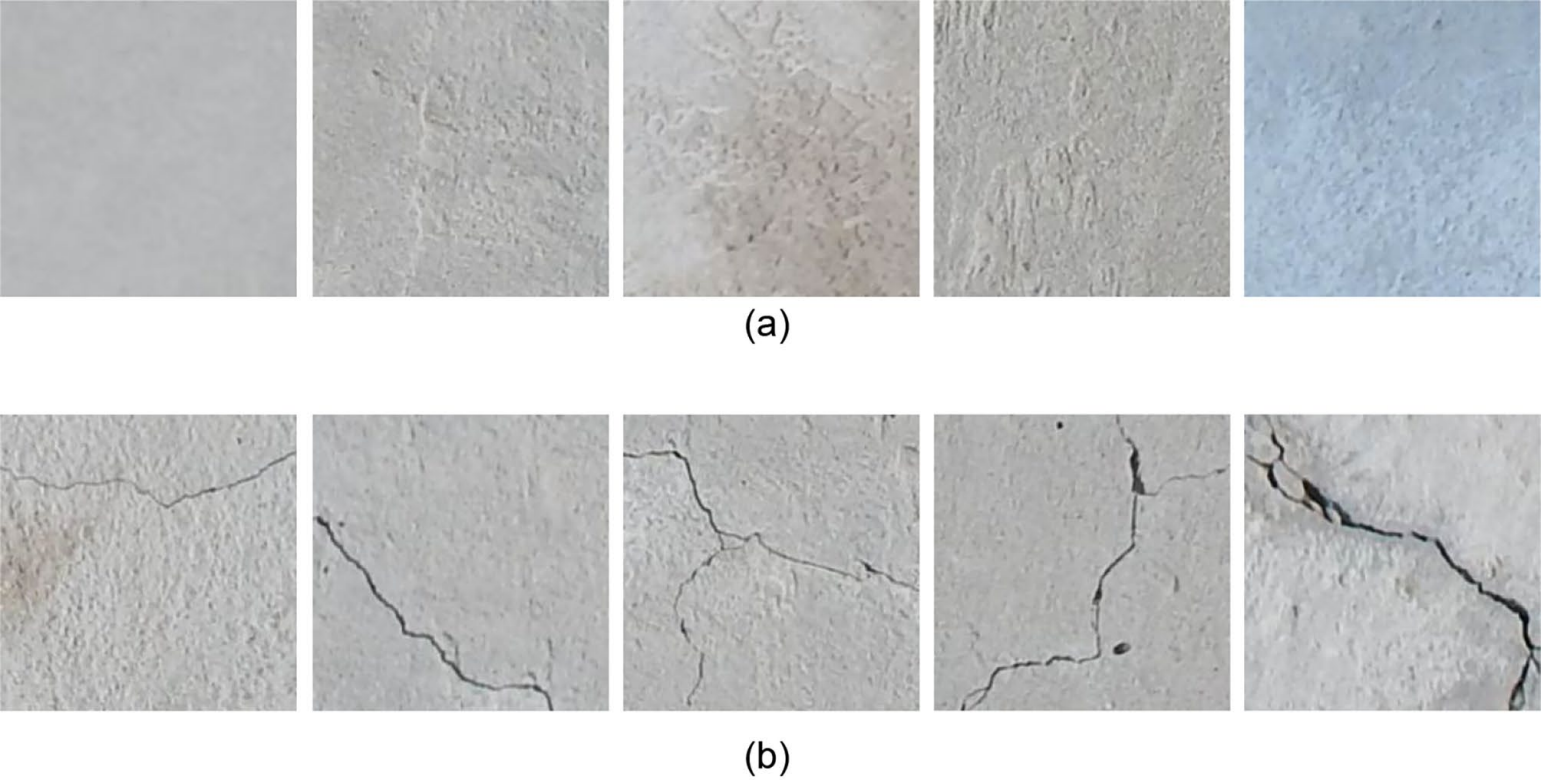
Original Crack Forest data: (**a**) Non-defective image. (**b**) Defective image.

**Figure 4:**
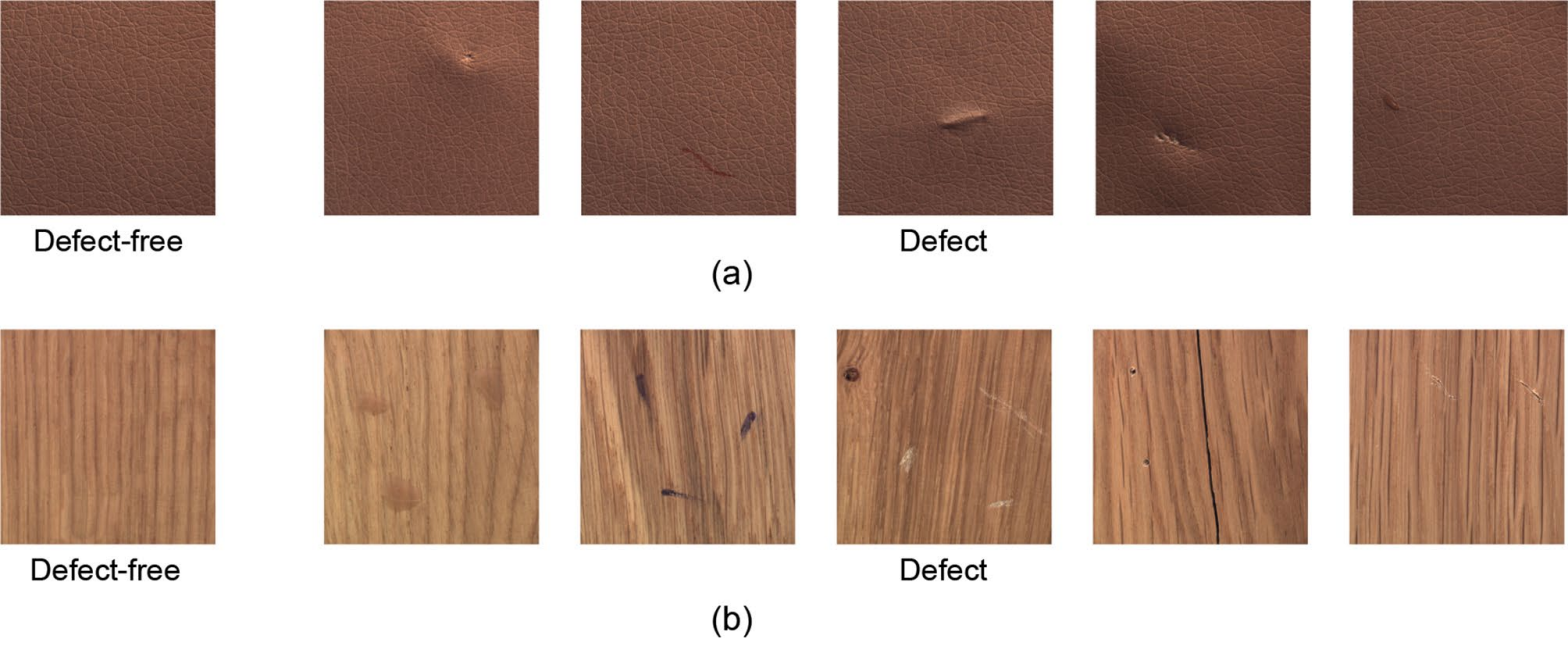
Original defective and non-defective MVTec data: (**a**) Leather image. (**b**) Wood image.

The defect image dataset used in this study is sourced from the publicly available Crack Forest Datasets by Northeastern University. This dataset focuses on defect detection in concrete roads. This dataset comprises various types of crack defects. Despite their presentation in different forms of cracks, all of them are categorized as a single type of defect in this paper. These defects result from various factors such as processes and equipment during the manufacturing production. It’s worth noting that defect sample images constitute only a minority portion of the dataset, reflecting the real-world data challenges faced in defect detection. To construct our experimental dataset, we categorize defect samples as one class and non-defective images as another class. The proposed model also tested the renowned public dataset MVTec, performing data augmentation on the leather and wood defect datasets. The construction of these datasets is similar in design to the CrackForest dataset. Tables 1 and 2 outline the specific construction of the datasets, while Figs. 3 and 4 showcase examples of various raw data samples.

**Table 1:** Crack Forest Dataset distribution.

	Defect-free	Defect
Train	1000	300
Test	330	60

**Table 2:** MVTec Dataset distribution.

	Defect-free	Defect
Train	227	65
Test	50	15

### Experiment implementation details

In the dataset of road crack defects, we validated the proposed image generation method (DG2GAN) and compared it with three related state-of-the-art generation methods: WGAN, D2GAN, and CycleGAN. We also compared it with the DG2GAN model that did not use DJS optimization (DG2GAN-no-DJS).

In all experiments of DG2GAN and DG2GAN-no-DJS, we maintained the same experimental parameter settings. In Formula (3), we set $\lambda$=0.8 and $\beta$=0.5. In Formula (4), we set $\mu$=0.05. $\lambda$ and $\beta$ are parameters that balance the adversarial loss of DG2 in two different directions. Parameter $\lambda$ controls the relative importance of the loss for the D1 series of discriminators, affecting the results of generating high-quality images. Parameter $\beta$ controls the relative importance of the loss for the D2 series of discriminators, influencing the diversity of the generated defect images. The values for both parameters typically range between 0 and 1. The setting of these two parameters requires a comprehensive consideration of the two different directional losses to find the balance point for the experimental objective and achieve the best generation effect. $\mu$ is the relative importance of the DJS loss, which, together with the traditional discriminator loss, jointly affects the discrimination results. This parameter can adjust the weight of the DJS loss in the total loss to avoid excessive influence on the training process. The setting of this value depends on the complexity of the training data distribution.

DG2GAN and CycleGAN were trained using the ADAM optimizer^37^. The batch size for DG2GAN was set to 8, while CycleGAN used a batch size of 4. WGAN and D2GAN had a batch size of 64. We trained DG2GAN and CycleGAN with an initial learning rate of 0.0002. The learning rate remained constant for the first 100 epochs and was linearly decayed to zero over the last 100 epochs. Due to the slower convergence of models without cycle consistency loss, the training epochs for WGAN and D2GAN were set to 2000. Each experiment was implemented on a computer equipped with 32GB RAM, Intel i9 CPU, NVIDIA GeForce RTX 3090 GPU and Ubuntu operating system.

### Time comparison

The training time cost of the defect generation model is an important metric that needs to be considered. The training time of a defect generation model is closely related to the network structure, the number of parameters, the training iterations, and the size of the dataset. This paper compares the training times of D2GAN, WGAN, CycleGAN, DG2GAN-no-DJS, and DG2GAN, as shown in Table 3. In the table, the training time represents the sum of the time spent on generating experiments. Although DG2GAN has more parameters and a longer training time compared to other models, the training time for DG2GAN is still acceptable. Furthermore, DG2GAN can generate defect images of higher quality and diversity compared to other methods.

**Table 3:** Comparison of training times of various image generation methods.

Methods	Variable size (Mb)	Epoch	Data volume	Training time (h)
D2GAN	12.64	1200	1300	4.81
WGAN	10.67	1200	1300	4.62
CycleGAN	26.30	200	1300	5.06
DG2GAN-no-DJS	31.72	200	1300	5.33
DG2GAN	32.32	200	1300	5.46

### Defect image generation

**Figure 5:**
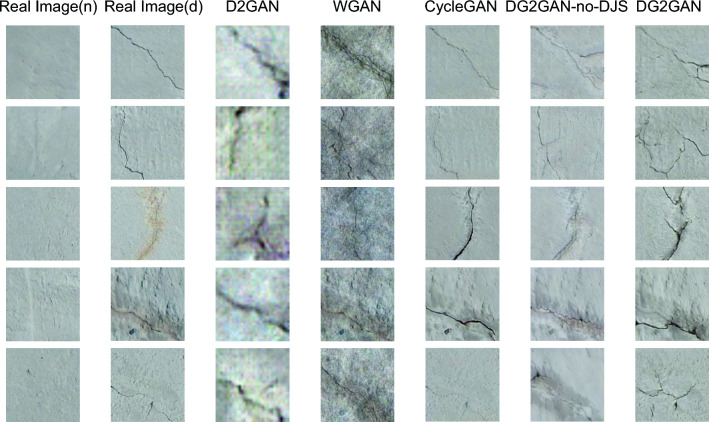
Defect images generated using various methods for the CrackForest dataset.

**Figure 6:**
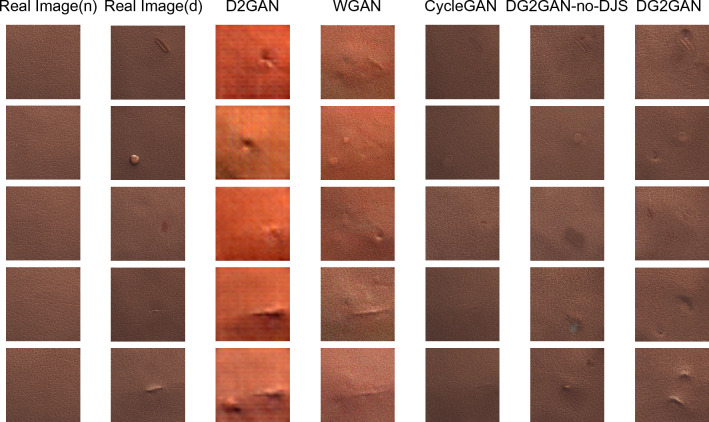
Defect images generated using various methods for the MVTec leather dataset.

**Figure 7:**
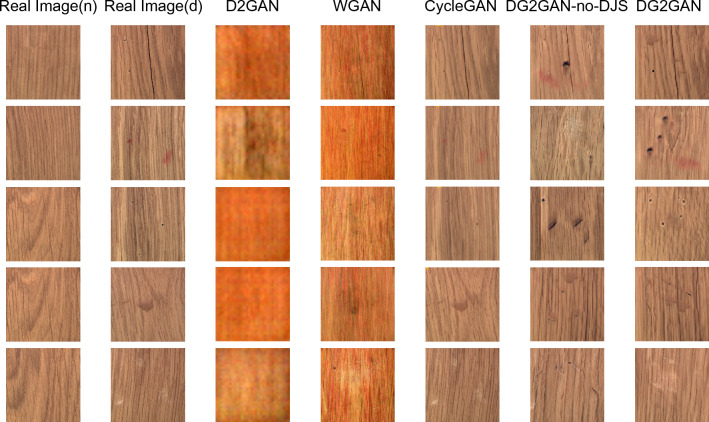
Defect images generated using various methods for the MVTec wood dataset.

In this paper, DG2GAN and other related generation methods were used to generate defect images on the CrackForest dataset and the MVTec dataset. Figures 5, 6, and 7 present the newly generated defect images for each method. The results demonstrate that the images generated by DG2GAN visually best meet the expectations for both high quality and diversity. Compared to WGAN and D2GAN, the images generated by DG2GAN and CycleGAN exhibit superior visual performance. This is because WGAN and D2GAN, which do not belong to feature transformation networks, do not utilize cycle consistency loss. By introducing additional noise, defect images generated by WGAN and D2GAN exhibit periodic noise, resulting in incomplete defect features and blurred defect-free regions, thereby compromising the authenticity of the data. In this paper, the introduction of DJS-optimized discriminator loss and the proposed DG2 adversarial loss yield superior generation results compared to models that do not use DJS optimization. This conclusion is validated both visually and through quantitative image evaluation metrics.

The introduction of adversarial loss and cycle consistency loss in image transformation addresses the imbalance in real production data and the scarcity of defect data. By using a large number of defect-free images to generate defective images, DG2GAN and CycleGAN ensure that the generated images closely resemble real image features in the defect-free regions. However, the strong consistency transformation structure of the CycleGAN model results in generated defect images that are essentially reproductions of the original data, failing to produce diverse defect images. In contrast, DG2GAN without DJS optimization (DG2GAN-no-DJS) exhibits inferior defect image quality compared to CycleGAN and DG2GAN. Although DG2GAN-no-DJS demonstrates some degree of diversity, its overall performance is significantly lower than that of DG2GAN. This indicates that without the inclusion of DJS-optimized discriminator loss and DG2 adversarial loss, the diversity and quality of the model cannot meet expectations. DG2GAN achieves proximity to the real image feature distribution in non-defective regions while presenting richer diversity in defective regions, showcasing more accurate defective features, less noise, and better boundaries. The generated defective images from DG2GAN exhibit almost no meaningless or erroneous data. Overall, DG2GAN excels in both quality and diversity. These conclusions are consistent in visual observation and evaluation metrics experiments, confirming that our proposed DG2GAN generation method demonstrates superior generative performance.

**Figure 8:**
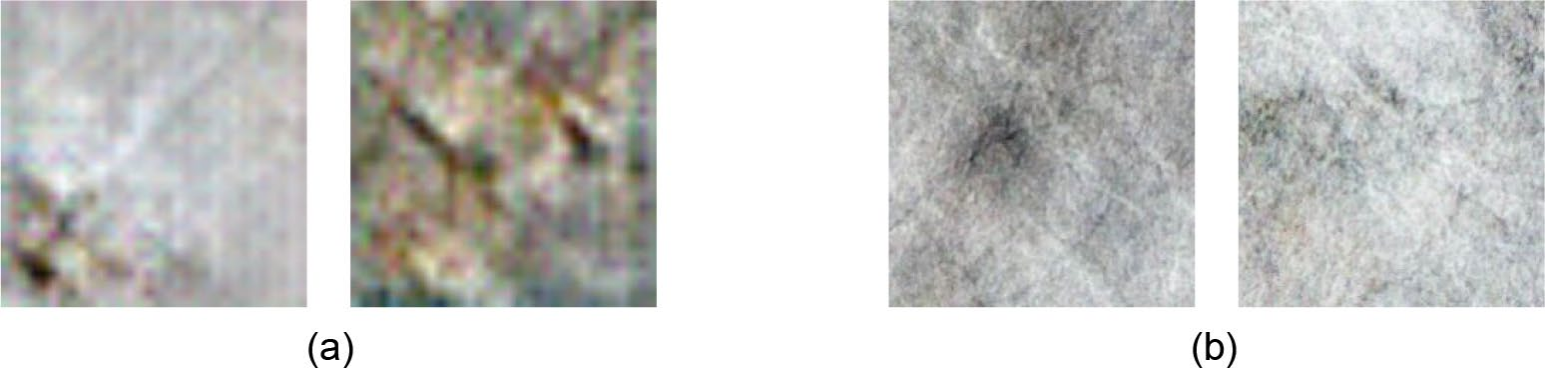
D2GAN and WGAN failed to generate defect images. (**a**) Generated image using D2GAN. (**b**) Generated image using WGAN.

We found that using the WGAN and D2GAN methods did not effectively generate defect images. Figure 8 illustrates examples where the WGAN and D2GAN methods failed to generate defect images. Specifically, images generated by WGAN resembled non-defective images and exhibited periodic noise, even when non-defective images were not present in the training dataset. On the other hand, defect images generated by D2GAN had significant noise, failing to accurately represent the features of the defect areas. This resulted in images lacking complete defect features, with non-defective areas appearing blurry. Without using cycle consistency loss, it is not possible to generate the expected defect images to address the existing issues of imbalanced data and scarcity of defect data. For models utilizing feature transformation such as DG2GAN and CycleGAN, similar failure cases did not occur. This outcome suggests that WGAN and D2GAN fail to extract defect features stably and accurately, resulting in poor image quality. Consequently, they are not suitable for generating data in scenarios with imbalanced and limited defect data.

### Quality evaluation of the GAN results

In addition to human visual evaluation, we also utilized the FID metric to compare the images generated by our proposed method with those generated by other advanced and related generative methods. This metric evaluated the overall performance of the generated images. The evaluation results are shown in Tables 4, 5, and 6.

**Table 4:** The CrackForest dataset was used to rate the overall performance metrics of images generated by various methods.

Methods	FID scores
D2GAN	224
WGAN	169
CycleGAN	87
DG2GAN-no-DJS	128
DG2GAN	**61**

Bold value is intended to highlight the experimental results of the method proposed.

**Table 5:** The MVTec leather dataset was used to rate the overall performance metrics of images generated by various methods.

Methods	FID scores
D2GAN	285
WGAN	189
CycleGAN	96
DG2GAN-no-DJS	102
DG2GAN	**70**

Bold value is intended to highlight the experimental results of the method proposed.

**Table 6:** The MVTec wood dataset was used to rate the overall performance metrics of images generated by various methods.

Methods	FID scores
D2GAN	287
WGAN	183
CycleGAN	87
DG2GAN-no-DJS	92
DG2GAN	**65**

Bold value is intended to highlight the experimental results of the method proposed.

The two noise-introducing generation methods, D2GAN and WGAN, exhibit different levels of performance in FID evaluation. D2GAN achieves the highest FID score, indicating the poorest overall performance of this model, with subpar image quality and diversity. The introduction of noise results in noisy generated images and fails to clearly express defect features. The FID score of WGAN is slightly lower than that of D2GAN, indicating that its generated images perform slightly better than those of D2GAN, but overall performance is still unsatisfactory compared to other generation methods. This highlights that the introduction of additional noise may increase diversity but has a detrimental effect on the quality of generated images, resulting in poor image generation performance in scenarios with imbalanced and limited defect data. Compared to these two methods, feature transformation models better address the issue of imbalanced and limited defect data. Among all methods, our proposed DG2GAN scores the lowest. However, when DG2GAN does not utilize DJS-optimized discriminator loss, its generated defect images receive a lower FID score than CycleGAN. This suggests that when DG2GAN does not use DJS optimization, the model tends to generate more diverse features but with unsatisfactory image quality, resulting in overall performance inferior to CycleGAN. However, the CycleGAN model prioritizes the quality of generated images while overlooking the diversity of generated sample features. With the introduction of DJS optimization, the overall performance of DG2GAN is improved, demonstrating better quality and diversity. Therefore, the optimization of DJS and the introduction of DG2 adversarial loss contribute to enhancing the quality and diversity of defect images generated by DG2GAN. Experimental results validate that our proposed DG2GAN model achieves the expected outcomes. Compared to other state-of-the-art generation methods, DG2GAN generates images with the lowest FID score, demonstrating that our proposed approach produces defect images with good quality and diversity in scenarios of data imbalance and limited defect data. It achieves a balance between high quality and high diversity, significantly improving defect classification performance.

### Data set expansion for resnet

The image generation method using a small number of defect samples was experimentally validated for the improvement of defect classification performance. Firstly, we applied our proposed image generation method to the CrackForest dataset and the MVTec dataset to enhance the defect image data. Secondly, we utilized the generated defect images to augment the training dataset of the classification model. The distribution of the augmented CrackForest data is shown in Table 7, and the distribution of the augmented MVTec data is shown in Table 8. Subsequently, the augmented training dataset was used to train the ResNet defect classification model. Finally, the original test set was employed to verify the accuracy and precision of the defect classification model, ensuring the consistency of detection. The same process was applied to other image generation methods, and for different defect image generation methods, we used the same ResNet classification model for classification recognition.

**Table 7:** Distribution of the augmented CrackForest dataset.

		Defect-free	Defect
Train	Real	1000	300
	Fake	0	600
Test		330	60

**Table 8:** Distribution of the augmented MVTec dataset.

		Defect-free	Defect
Train	Real	227	65
	Fake	600	800
Test		50	15

**Figure 9:**
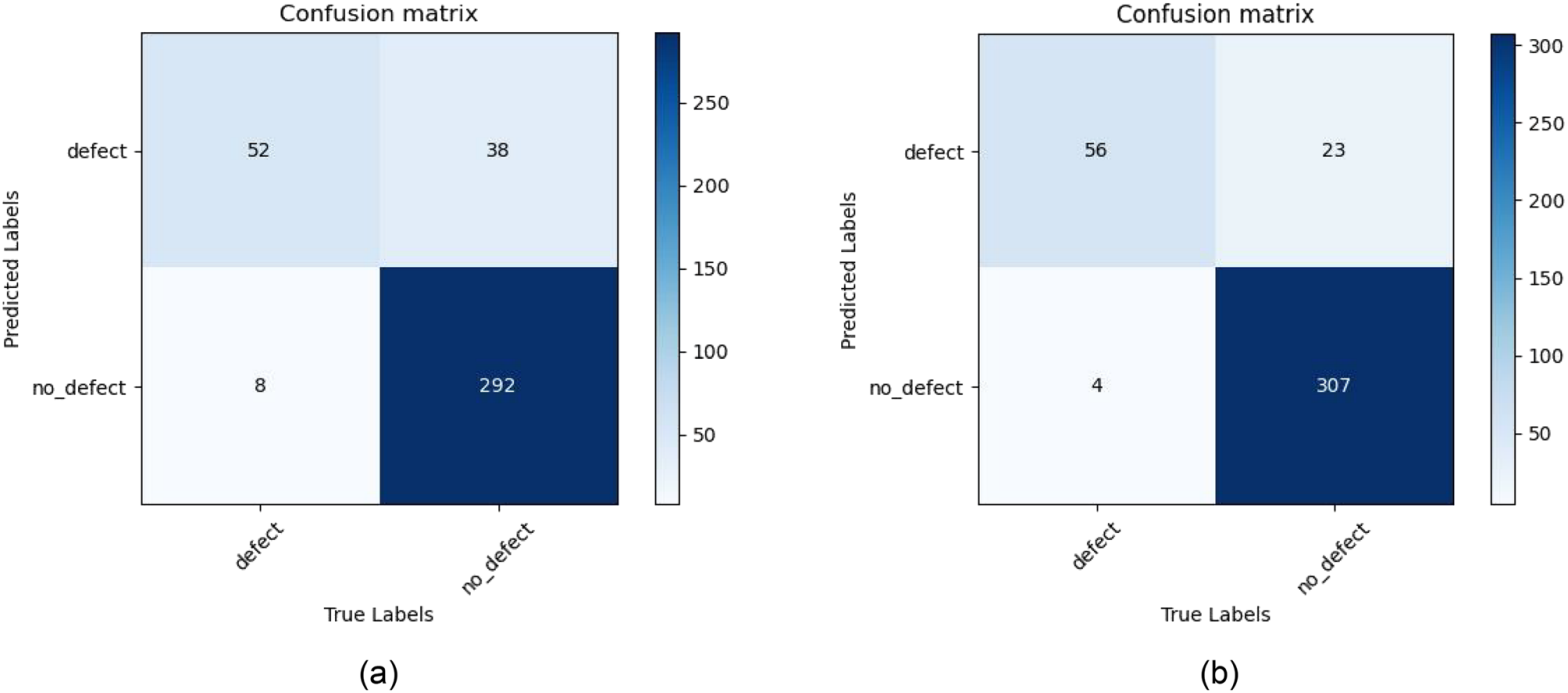
CrackForest dataset confusion matrix. (**a**) Resnet-only confusion matrix. (**b**) DG2GAN + Resnet confusion matrix.

Expanding the dataset using various image generation methods, the defect classification model is trained on the augmented training set, with the ResNet classification model set to run for 1000 epochs. Tables 9 and 10 present the accuracy and precision of classification and recognition after data augmentation using different model generation methods on the CrackForest dataset. It is evident that whether using ResNet34 or ResNet50 for recognition, the detection performance is relatively poor when only the original data is used for defect classification and recognition. The data augmentation methods, D2GAN and WGAN, which introduce noise, show some improvement in detection performance, but the effect is moderate. The enhancement of detection performance is more significant when using image transformation as the augmentation method, particularly with the DG2GAN generation method proposed in this paper, which yields the best detection results after enhancing the data. Figure 9 displays the confusion matrix for ResNet-only and DG2GAN+ResNet, illustrating the computational results of classification recognition using our proposed generation method to expand the data under the recognition model. The same conclusion can be validated with the MVTec dataset after data augmentation for classification model recognition. Tables 11 and 12 present the recognition results using ResNet50 on the MVTec dataset after data augmentation with different model generation methods. These results are consistent with those obtained from the CrackForest dataset. From this, we can draw the following conclusions.

1. Due to the imbalance, lack of diversity, and insufficient quantity of defective and non-defective images in the original data, the detection model could not converge stably, resulting in significant oscillations when the dataset was not augmented. Simultaneously, this led to low accuracy and precision in defect classification recognition. After employing various data augmentation methods on the dataset, the accuracy and precision of the defect classification recognition model improved significantly. Particularly, utilizing the DG2GAN method proposed in this paper for data augmentation enhanced the stability of the defect classification recognition model, resulting in the best performance in terms of accuracy and precision.
2. The model trained on the expanded dataset is more robust and better able to resist the impact of dirty and invalid data. When using WGAN and D2GAN for dataset augmentation, the images that are not successfully generated are not rejected. Although training a defect classification model with datasets augmented using these two generation methods may slightly improve accuracy, the improvement is not significant. This is because these two generation methods, WGAN and D2GAN, cannot effectively meet the requirements of expanding the dataset. The diversity of defect images generated by noise may introduce irrelevant transformations, compromising the authenticity of the data and generating some unwanted and meaningless diverse defect images. Because CycleGAN uses cycle-consistent networks, its accuracy and precision in classification models are slightly better than those of WGAN and D2GAN, but the diversity of images it generates is poor. DG2GAN-no-DJS generates some diverse images, but its image quality and overall performance are relatively poor. Therefore, the difference in accuracy and precision in detection between CycleGAN and DG2GAN-no-DJS is not significant. Both of them have inferior overall performance compared to DG2GAN, resulting in much lower accuracy and precision in classification recognition.
3. Among all the methods mentioned above, the defect classification recognition model trained on the extended dataset generated by DG2GAN performs the best. For the CrackForest dataset, the accuracy increased from 86.9 to 94.6%, and the classification recognition precision surpassed other generation methods, rising from 59.8 to 80.2%. Similarly, for the MVTec dataset, both accuracy and precision were improved under data augmentation with the DG2GAN model. Compared to other image generation methods in this paper, the defect images generated by DG2GAN exhibit superior overall performance, featuring higher image quality and diversity. They accurately and comprehensively reflect the authentic characteristics of the defect dataset, contributing to the enhancement of defect detection and recognition performance.

**Table 9:** The recognition accuracy and precision of different generation methods under the ResNet34 model after data augmentation for the CrackForest dataset.

Methods		Accuracy	Precision
Resnet-only	Defect	86.7%	0.578
	Defect-free	88.5%	0.973
D2GAN+Resnet	Defect	88.1%	0.684
	Defect-free	92.7%	0.978
WGAN+Resnet	Defect	88.3%	0.616
	Defect-free	90%	0.977
CycleGAN+Resnet	Defect	90%	0.628
	Defect-free	90.3%	0.98
DG2GAN-no-DJS+ Resnet	Defect	91.7%	0.647
	Defect-free	90.9%	0.984
DG2GAN+ Resnet	Defect	**93.**3%	**0.709**
	Defect-free	**93**%	**0.987**

Bold value is intended to highlight the experimental results of the method proposed.

**Table 10:** The recognition accuracy and precision of different generation methods under the ResNet50 model after data augmentation for the CrackForest dataset.

Methods		Accuracy (%)	Precision
Resnet-only	Defect	86.9	0.598
	Defect-free	88.9	0.978
D2GAN+Resnet	Defect	88.8	0.689
	Defect-free	93	0.981
WGAN+Resnet	Defect	88.9	0.621
	Defect-free	90.3	0.979
CycleGAN+Resnet	Defect	90.9	0.635
	Defect-free	90.8	0.982
DG2GAN-no-DJS+ Resnet	Defect	92	0.651
	Defect-free	91.4%	0.986
DG2GAN+ Resnet	Defect	**94.6**	**0.802**
	Defect-free	**94.5**	**0.99**

Bold value is intended to highlight the experimental results of the method proposed.

**Table 11:** The recognition accuracy and precision of different generation methods under the ResNet34 model after data augmentation for the MVTec leather dataset.

Methods		Accuracy (%)	Precision
Resnet-only	Defect	82.1	0.51
	Defect-free	83.8	0.681
D2GAN+Resnet	Defect	85.7	0.673
	Defect-free	87.5	0.724
WGAN+Resnet	Defect	87.8	0.706
	Defect-free	88.3	0.742
CycleGAN+Resnet	Defect	89.7	0.772
	Defect-free	91.6	0.818
DG2GAN-no-DJS+ Resnet	Defect	90.3	0.785
	Defect-free	92.1	0.824
DG2GAN+ Resnet	Defect	**92.6**	**0.804**
	Defect-free	**92.8**	**0.856**

Bold value is intended to highlight the experimental results of the method proposed.

**Table 12:** The recognition accuracy and precision of different generation methods under the ResNet34 model after data augmentation for the MVTec wood dataset.

Methods		Accuracy (%)	Precision
Resnet-only	Defect	83.6	0.54
	Defect-free	84.4	0.688
D2GAN+Resnet	Defect	86.7	0.68
	Defect-free	87.8	0.731
WGAN+Resnet	Defect	87.5	0.734
	Defect-free	88.9	0.766
CycleGAN+Resnet	Defect	90.3	0.784
	Defect-free	91.9	0.828
DG2GAN-no-DJS+ Resnet	Defect	90.8	0.796
	Defect-free	92.5	0.835
DG2GAN+ Resnet	Defect	**93.1**	**0.826**
	Defect-free	**93.8**	**0.887**

Bold value is intended to highlight the experimental results of the method proposed.

**Figure 10:**
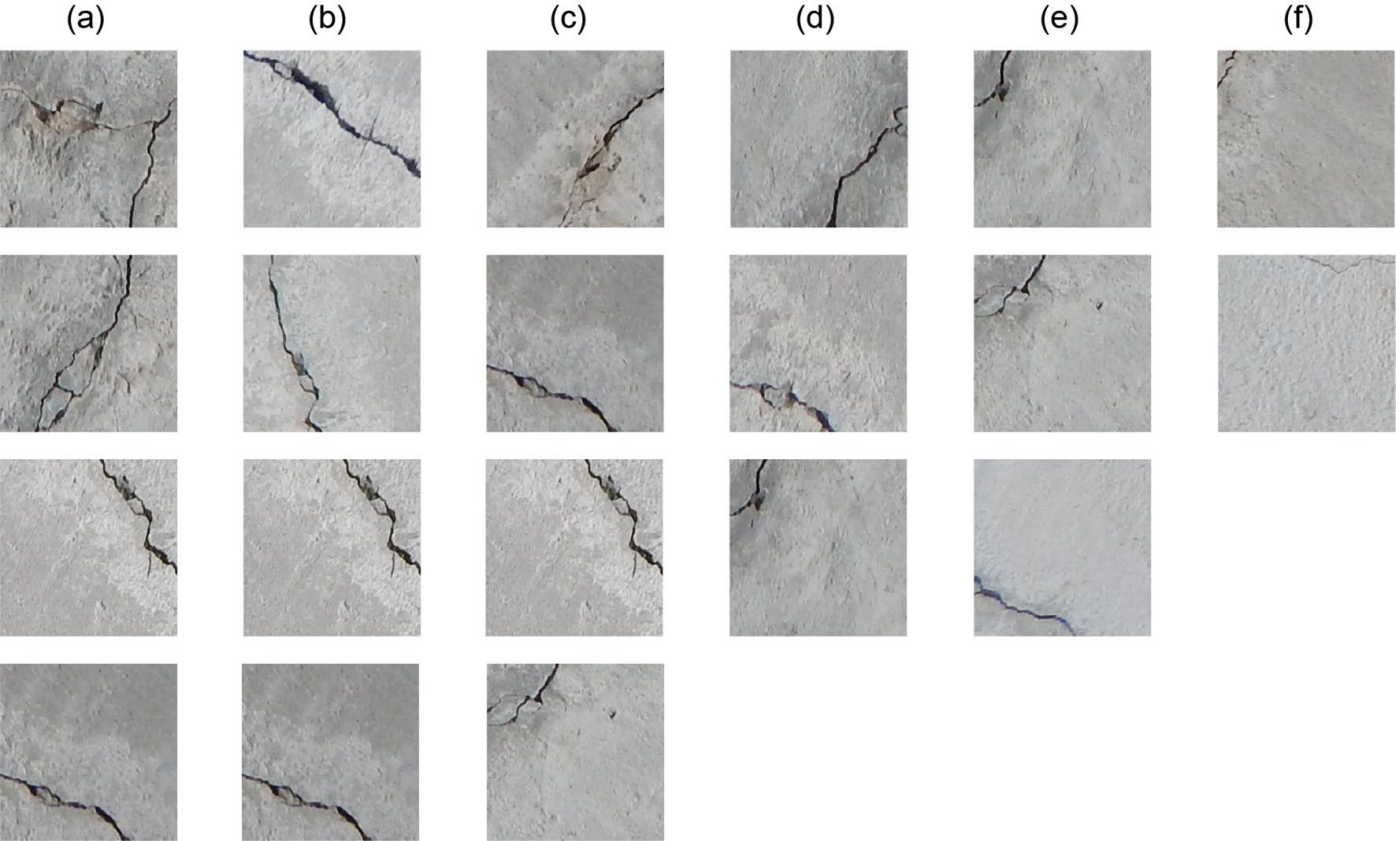
Defect image that various kinds of methods fail to recognize. (**a**) Resnet-only. (**b**) D2GAN+Resnet. (**c**) WGAN+Resnet. (**d**) CycleGAN+Resnet. (**e**) DG2GAN-no-DJS+Resnet. (**f**) DG2GAN+Resnet.

Analyzing the images misclassified by the detection model provides insights into whether data augmentation improves deep learning for surface defect recognition technology. As shown in Fig. 10, defect images misclassified after data augmentation using different generation methods, we can draw the following conclusions based on these results.

1. The Resnet-only model is unable to recognize the defects in (a). The reason lies in the imbalance of the original data, where there is a scarcity of defect samples. When using the Resnet model for recognition, the lack of sufficient defect samples in the original data, especially for certain specific types of defect images, prevents the model from learning enough information for accurate classification.
2. Compared to the Resnet-only model trained solely on the original data, the defect recognition with different data augmentation methods has shown an improvement in misidentification cases. D2GAN+Resnet and WGAN+Resnet have shown improvements in misidentification compared to training solely with original data. Misidentifications are more focused on small crack defects, and the probability of identifying these defects as non-defective is decreasing. However, with the data augmentation methods of CycleGAN+Resnet and DG2GAN-no-DJS+Resnet, as the diversity and quality of the data increase, the number of misidentified defects is decreasing. Large crack defects are no longer misidentified, and the probability of previously misidentifying small crack defects as non-defective is continuously decreasing.
3. Compared to the Resnet-only model and other generation methods, DG2GAN + Resnet significantly improves the model’s misidentification. This is evident both in terms of the number of misidentified images and the types of defects, demonstrating that the defect images generated by our proposed method align with expectations. The images generated by this method exhibit the best overall performance among all methods and contribute to enhancing defect detection and recognition performance.

## Discussion

The model proposed in this paper is based on a cyclic network structure, making it susceptible to the influence of strong consistency transformations from cycle-consistency loss. In initial experiments, it is difficult to achieve the optimal balance of weight parameters, resulting in generally poor diversity of the generated images. During experimental adjustments, the overall performance may be suboptimal, manifesting in two ways: first, the loss parameters and weight adjustments tend to generate high-quality images, leading to poor image diversity; second, the loss parameters and weight adjustments tend to generate more diverse samples, leading to lower image quality. Experimental analysis is needed to verify that the set parameters achieve a balance between quality and diversity, resulting in optimal overall performance of the generated images. However, the model mostly generates images with obvious defects and is not yet mature in generating images with minor defects. Therefore, future research needs to focus on implementing unsupervised data augmentation to enhance the model’s ability to generate small defects. Nonetheless, under the current data conditions, experiments have shown that this model has already achieved high-quality and high-diversity data augmentation, improving the detection accuracy of surface defect detection models and promoting the success of surface defect detection tasks.

In our experiments, GAN-based data augmentation methods significantly improved the accuracy and precision of defect classification and recognition. This improvement is mainly attributed to achieving data balance through the expansion of training data, resulting in enhancements in data distribution and the training process. Firstly, the data augmentation method using DG2GAN expands the training dataset, achieving a balance between defective and non-defective data. This helps the model learn sufficient features from both defective and non-defective samples, rather than solely focusing on one type of feature, which is crucial for improving classification and recognition performance. Secondly, it improves data distribution, where data augmentation makes the boundary between defective and non-defective data clearer. This aids the model in better identifying data in boundary regions, especially for data with fewer defect features. Finally, based on GAN-based data augmentation, the diversity and completeness of the data distribution during the training process are enhanced, reducing the risk of overfitting and consequently improving recognition accuracy and precision. Therefore, the model proposed in this paper addresses situations where data is imbalanced and defect data is scarce in defect detection. This model can perform data augmentation, thereby enhancing the performance of defect detection and recognition.

## Conclusions

In this paper, addressing the specific circumstances and challenges of manufacturing defect datasets, we propose a novel image generation method called DG2GAN. By introducing a dual-discriminator framework and leveraging cycle consistency loss to generate defect images based on defect-free images, we tackle the issue of insufficient diversity in generated images. In the dual-discriminator framework, we incorporate DJS optimized discriminator loss to encourage the generation of diverse defect images. Furthermore, we design a new DG2 adversarial loss to balance the quality and diversity of the generated defect images, achieving high-quality and diversified defect data augmentation. The experimental results indicate that augmenting the publicly available defect dataset, Crack Forest Datasets from Northeastern University, using the DG2GAN method significantly improves the defect classification recognition performance compared to other state-of-the-art image generation algorithms. Specifically, the defect recognition accuracy increases from 86.9% to 94.6%, and the precision increases from 59.8 to 80.2%. Compared to Crack Forest Datasets enhanced by other generation algorithms, the dataset enhanced by DG2GAN exhibits higher accuracy and precision. To validate the effectiveness of the proposed model, the MVTec dataset was also subjected to the same data augmentation. Recognition experiments were conducted using the augmented data, and the results were consistent with the previous conclusions. These results demonstrate that our proposed DG2GAN generation method offers significant advantages in addressing the limitations of limited defect data in manufacturing industries and improving defect classification recognition under conditions of imbalanced and limited defect data. However, the aforementioned research indicates that this model requires experimental analysis to determine the optimal weights for each type of training data. Additionally, the recognition experiment conclusions suggest that the model’s ability to generate small defects needs improvement. Therefore, it can be concluded that the model has a certain level of training difficulty and is not yet mature in generating small defects. Therefore, future research can focus on improving the model’s stability and its ability to generate small defects, further enhancing the model’s generalization and robustness. Additionally, addressing data issues in detection and recognition through data augmentation will be a key area of focus.

## Data Availability

All data for the experiments used in this study are available via the web. Hyperlinks at the bottom provide links to the datasets. Crack Forest dataset: https://github.com/cuilimeng/CrackForest. MVTec dataset: https://www.mvtec.com/company/research/datasets.
